# Neurofibroma Within a Nevus Sebaceus: A Case Report

**DOI:** 10.7759/cureus.28645

**Published:** 2022-08-31

**Authors:** Dylan Maldonado, Frances Hanson, Heather Layher, Michelle Tarbox

**Affiliations:** 1 Department of Dermatology, Texas Tech University Health Sciences Center, Lubbock, USA; 2 Medicine, Texas Tech University Health Sciences Center Paul L. Foster School of Medicine, Lubbock, USA

**Keywords:** ras gene mutations, k-ras, -dermatopathology, solitary neurofibroma, nevus sebaceous

## Abstract

Nevus sebaceus most commonly presents as a yellow, alopecic plaque on the head or neck in childhood and evolves into a verrucous plaque at puberty. Numerous secondary tumors may arise within nevus sebaceus lesions. Tumors of mesenchymal origin have been rarely documented. We present a unique case of a patient who presented with a nevus sebaceus on the scalp. Excision of the tumor and subsequent histopathology of the lesion revealed a nevus sebaceus with a desmoplastic trichilemmoma, a tumor of follicular infundibulum, and a neurofibroma. This case highlights a rare finding of a mesenchymal tumor, and the first reported neurofibroma, arising in association with a nevus sebaceus.

## Introduction

Nevus sebaceus is a hamartoma of the skin, and it comprises multiple skin elements including the epidermis, hair follicles, sebaceous units, apocrine units, and eccrine units, as well as mesenchymal components [1]. Nevus sebaceus most commonly presents as a yellow, alopecic plaque, predominately on the face and scalp, and evolves into a velvety, mamillated, verrucous plaque, most often during puberty [1,2]. Secondary tumors developing within nevus sebaceus lesions are associated with advanced age, with the vast majority occurring in adulthood [3]. The most prevalent secondary benign neoplasm arising from nevus sebaceus is trichoblastoma, whereas basal cell carcinoma is the most common secondary malignant neoplasm [2-3]. The rare finding of multiple distinct tumors arising within nevus sebaceus may offer insights into the mechanisms and cell lineages involved in secondary transformations arising from nevus sebaceus. We present a case of a neurofibroma arising in association with a nevus sebaceus along with a tumor of the follicular infundibulum and desmoplastic trichilemmoma.

## Case presentation

**Figure 1:**
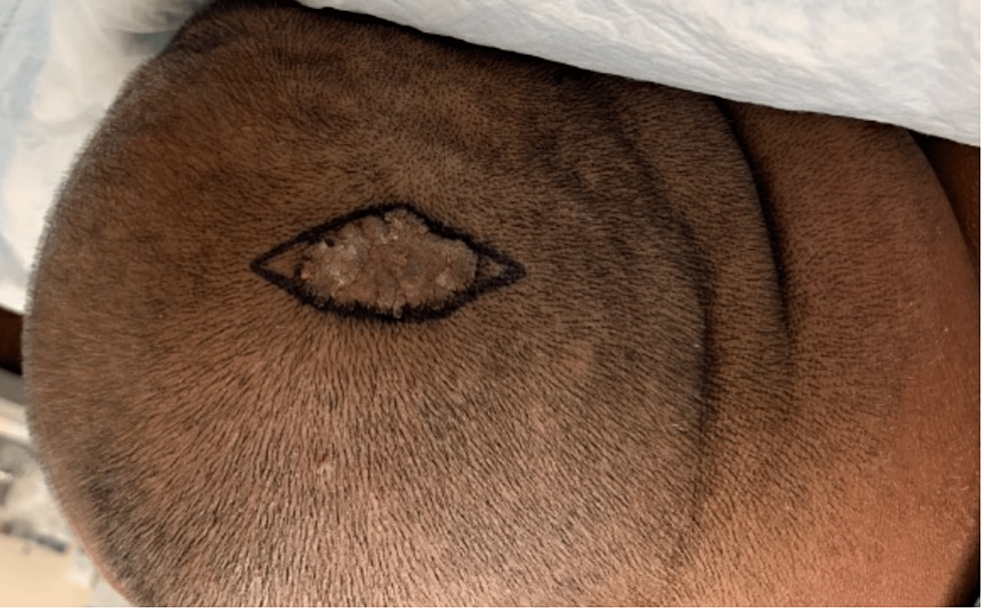
30 x 15-mm mamillated pink plaque on the patient's left vertex scalp with demarcated incisional margin

A 46-year-old male presented to the dermatology clinic with a lesion on the scalp. The patient reported that this lesion had been present since he was a child and had not undergone any recent change. Physical examination revealed a 30 x 15-millimeter, linear, mamillated, tan-to-pink plaque consistent with the diagnosis of nevus sebaceus (Figure 1).

**Figure 2:**
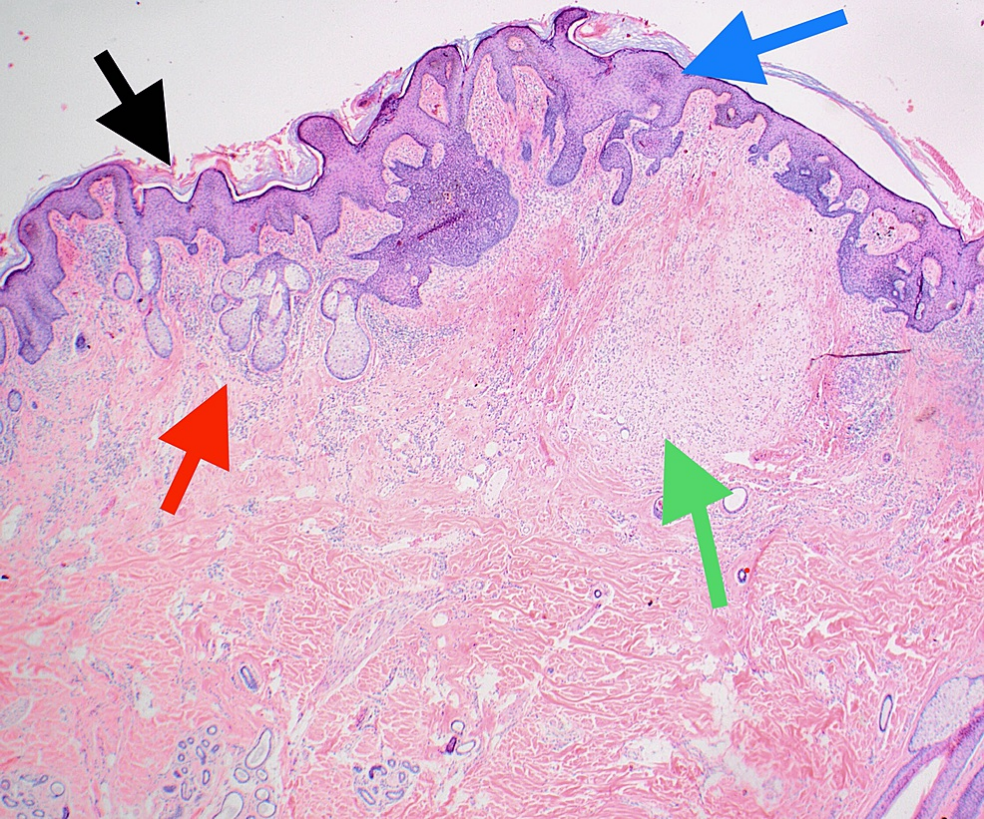
This image shows epidermal papillomatosis (black arrow) with numerous large sebaceous glands (red arrow) directly attached to the overlying acanthotic epidermis (blue arrow) consistent with a nevus sebaceus. Neurofibroma is seen (green arrow) Hematoxylin and eosin (H&E) staining, 20x magnification

Complete elliptical excision was performed at a subsequent visit with narrow margins. The histopathology of the lesion displayed epidermal papillomatosis with numerous large sebaceous glands directly attached to the overlying acanthotic epidermis consistent with a nevus sebaceus (Figure 2).

**Figure 3:**
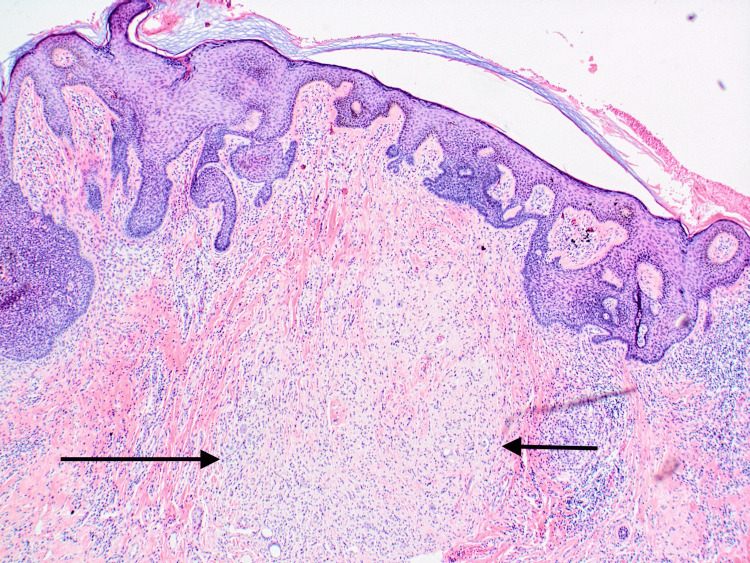
A proliferation of bland-appearing spindled cells within the dermis surrounded by loose collagenous stroma is noted, consistent with neurofibroma (black arrows) in conjunction with the nevus sebaceus H&E staining, 40x magnification

**Figure 4:**
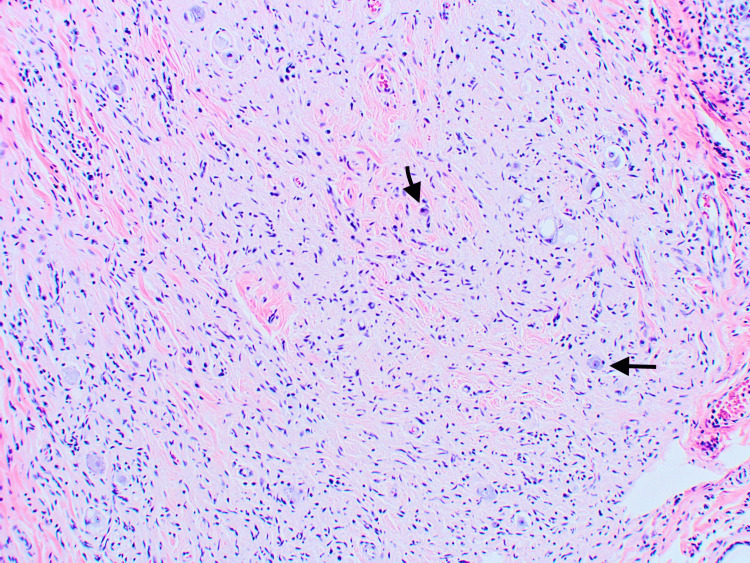
Higher power view of the bland-appearing spindled cells within the dermis surrounded by loose collagenous stroma and admixed mast cells (noted by arrows) was identified, consistent with a neurofibroma H&E staining, 100x magnification

A proliferation of bland-appearing spindled cells within the dermis surrounded by loose collagenous stroma and admixed mast cells was identified, consistent with a neurofibroma (Figures 3, 4).

**Figure 5:**
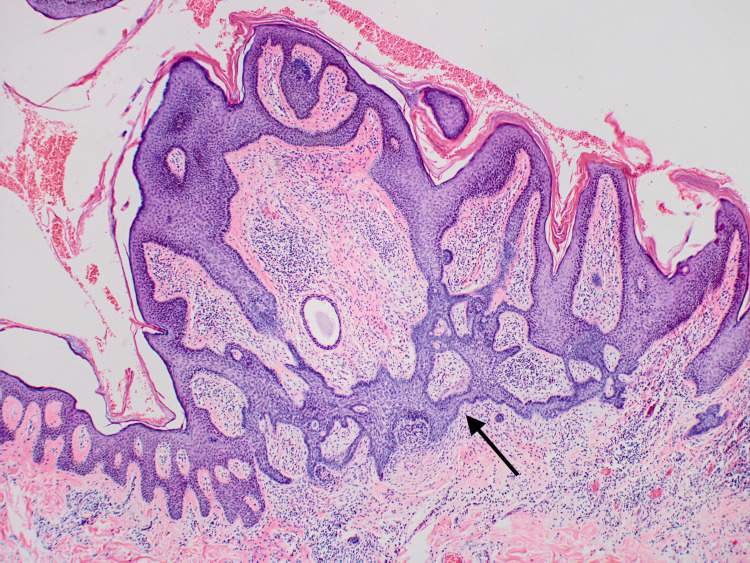
Tumor of follicular infundibulum demonstrating inter-anastomosing strands of the basaloid epithelium (arrow) emerging from the underside of the epidermis H&E staining, 40x magnification

**Figure 6:**
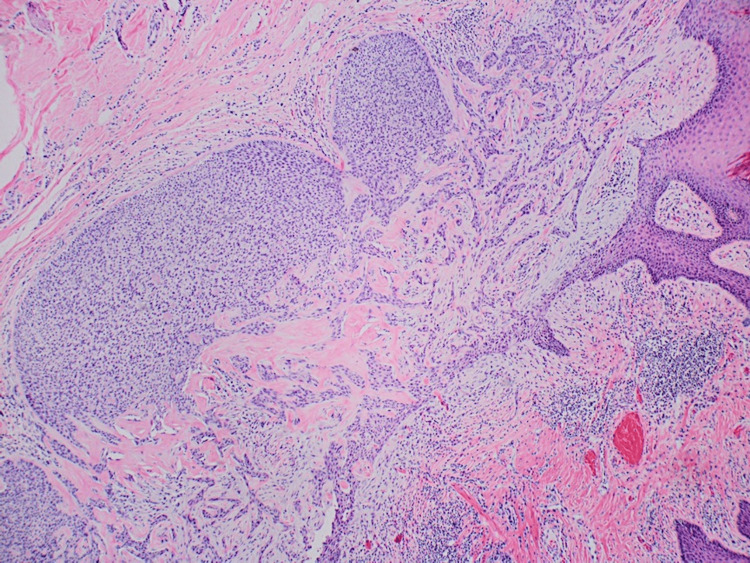
Desmoplastic trichilemmoma demonstrating peripheral palisade of nuclei surrounded by a hyaline cuticle with areas of desmoplasia H&E staining, 40x magnification

Of note, a desmoplastic trichilemmoma was noted to include a peripheral palisade of nuclei surrounded by a hyaline basement membrane with areas of desmoplasia. Finally, a follicular neoplasm composed of inter-anastomosing strands of the basaloid epithelium was found emerging from the underside of the epidermis, consistent with a tumor of the follicular infundibulum. The pathologic diagnosis for the lesion was a nevus sebaceus with a neurofibroma, a tumor of the follicular infundibulum (Figure 5), and a desmoplastic trichilemmoma (Figure 6). There was no evidence of recurrence of the nevus sebaceus at the follow-up visit one year later.

## Discussion

Nevus sebaceus lesions develop as the result of disorderly organization in epidermal, follicular, sebaceous, and apocrine structures. The neoplastic potential of nevus sebaceus can be explained by the pluripotency of the primary epithelial germ cells, which give rise to these distinct epithelial, sebaceous, and apocrine structures [4]. As such, multiple tumors are capable of developing within a nevus sebaceus due to the proliferation of pluripotent epithelial germ cells that each follow distinct lineages [4]. The development of nevus sebaceus has been related to mosaic mutations in "Harvey rat sarcoma virus", more commonly referred to as HRAS, and "Kristen rat sarcoma virus" or KRAS [5]. These mutations lead to the activation of mitogen-activated protein kinase and the phosphoinositide 3-kinase pathway and increased cellular proliferation [5-6]. The resulting "RASopathy" explains the potential for cellular proliferation within nevus sebaceus lesions and the development of secondary neoplasms [7]. Even accounting for pluripotency and mosaic RAS (rat sarcoma) mutations, which can promote a variety of cell lineages to proliferate, the occurrence of multiple benign and malignant tumors arising within the same nevus sebaceus remains a rare finding.

Tumors of mesenchymal origin are rarely associated with nevus sebaceus. There are rare reports of a piloleiomyoma, leiomyosarcoma, and adenomyoepithelioma arising within or in association with nevus sebaceus [2,8-9]. The authors could not find any reported cases of neurofibromas in association with nevus sebaceus. However, there is a reported case of segmental neurofibromatosis with a nevus sebaceus within the same distribution [10]. Nevus sebaceus lesions have also been associated with neurocutaneous syndromes leading some authors to hypothesize that neurofibromatosis and nevus sebaceus, at some point in their development, share similar tissues of neural origin [11]. As noted above, HRAS and KRAS mutations have been identified and nevus sebaceus has been classified as a "RASopathy" [6]. Similarly, neurofibromatosis 1 has been classified as a “RASopathy" [10]. Whether the neurofibroma in our case had an underlying molecular alteration, similar to that of a nevus sebaceus, leading to its presence, or whether it was an incidental finding remains uncertain but is an important association to consider.

## Conclusions

We presented a rare finding of a neurofibroma, a tumor of mesenchymal origin, arising in association with a nevus sebaceus along with a tumor of the follicular infundibulum and desmoplastic trichilemmoma. This is a unique finding and could help further characterize the development of secondary tumors in nevus sebaceus neoplasms.
